# EXPLORING EPIGENETIC CLOCKS AND COGNITIVE CHANGE IN MIDDLE ADULTHOOD

**DOI:** 10.1093/geroni/igae098.4182

**Published:** 2024-12-31

**Authors:** Daisy Zavala, Zita Oravecz, Jennifer Graham-Engeland, Christopher Engeland, Martin Sliwinski, Krishna Veeramah, Stacey Scott

**Affiliations:** Stony Brook University, Stony Brook, New York, United States; The Pennsylvania State University, University Park, Pennsylvania, United States; The Pennsylvania State University, University Park, Pennsylvania, United States; The Pennsylvania State University, University Park, Pennsylvania, United States; The Pennsylvania State University, University Park, Pennsylvania, United States; Stony Brook University, Stony Brook, New York, United States; Stony Brook University, Stony Brook, New York, United States

## Abstract

Positive age acceleration, in which a person’s epigenetic age is older than their chronological age, was associated with worse mean-level cognitive performance and higher moment-to-moment intraindividual variability in our prior work utilizing only a single burst of data. The present study extends this work by utilizing a Bayesian Double Exponential Model (BDEM) to examine longitudinal changes in cognition while accounting for practice effects. In a diverse sample of 142 midlife adults, baseline age acceleration was derived from five DNA methylation epigenetic clocks (Horvath 1, Horvath 2, Hannum, PhenoAge, GrimAge). Across four annual bursts of data, ambulatory processing speed and working memory was determined through the symbol search and n-back task, respectively. Using the BDEM framework, for both tasks we found that peak performance (at burst 1) was slower for individuals with greater age acceleration and those who were chronologically older. However, we found no longitudinal changes in peak performance on average, and neither age acceleration nor chronological age were predictive of differences in longitudinal change. Interestingly, greater age acceleration (but not chronological age) was associated with more fluctuations in scores across the study period, but greater chronological age (not age acceleration) was linked with more forgetting between bursts in the symbol search task. Although it is unclear what is driving opposing findings, these results reveal that even in an age heterogeneous sample spanning midlife (prior to expected cognitive declines, or onset of impairment) differences in age acceleration predict differences in peak performance and variability.

